# Feature Extraction and Matching of Humanoid-Eye Binocular Images Based on SUSAN-SIFT Algorithm

**DOI:** 10.3390/biomimetics8020139

**Published:** 2023-03-29

**Authors:** Xiaohua Xia, Haoming Xiang, Yusong Cao, Zhaokai Ge, Zainan Jiang

**Affiliations:** 1 Key Laboratory of Road Construction Technology and Equipment of MOE, Chang’an University, Xi’an 710064, China; 2 State Key Laboratory of Robotics and System, Harbin Institute of Technology, Harbin 150001, China; jiangzainan@hit.edu.cn

**Keywords:** humanoid-eye binocular images, feature matching, multi-scale, SUSAN, SIFT

## Abstract

Imitating the visual characteristics of human eyes is one of the important tasks of digital image processing and computer vision. Feature correspondence of humanoid-eye binocular images is a prerequisite for obtaining the fused image. Human eyes are more sensitive to edge, because it contains much information. However, existing matching methods usually fail in producing enough edge corresponding pairs for humanoid-eye images because of viewpoint and view direction differences. To this end, we propose a novel and effective feature matching algorithm based on edge points. The proposed method consists of four steps. First, the SUSAN operator is employed to detect features, for its outstanding edge feature extraction capability. Second, the input image is constructed into a multi-scale structure based on image pyramid theory, which is then used to compute simplified SIFT descriptors for all feature points. Third, a novel multi-scale descriptor is constructed, by stitching the simplified SIFT descriptor of each layer. Finally, the similarity of multi-scale descriptors is measured by bidirectional matching, and the obtained preliminary matches are refined by subsequent procedures, to achieve accurate matching results. We respectively conduct qualitative and quantitative experiments, which demonstrate that our method can robustly match feature points in humanoid-eye binocular image pairs, and achieve favorable performance under illumination changes compared to the state-of-the-art.

## 1. Introduction

Visual information capture and processing are of significant scientific importance in the field of modern engineering technology, but visual functions performed by existing vision systems are still quite different to those of human vision. Because of the remarkable ability of human vision, analyzing and imitating the visual characteristics of human eyes can enrich the theoretical basis of the computer vision and advance the development of machine vision technology [1]. As one of the essential challenges in computer vision, constructing reliable correspondences between two images containing the same or similar scenes is a fundamental problem, and it acts as a key prerequisite for a wide spectrum of tasks including SLAM, image registration, image fusion, and image mosaic [2,3]. Although image matching algorithms have been well studied, it is still rare to effectively introduce human visual characteristics into the matching process, and challenging to robustly match humanoid-eye binocular images with significant viewpoint and view direction differences.

**Figure 1:**
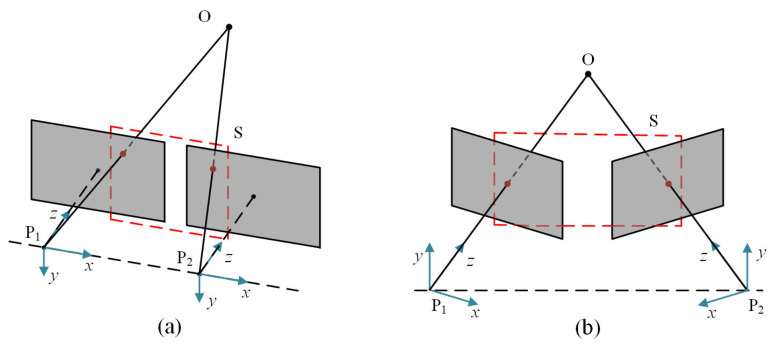
Two kinds of binocular camera model. (**a**) Conventional parallel binocular camera model. (**b**) Humanoid-eye convergent binocular camera model. The red boxes stand for the overlapping area. Compared with the parallel binocular model, images captured by the convergent binocular model have wider overlapping areas.

In general binocular vision models, since just a translation variation exists between two cameras, the corresponding points in the overlapping area only have horizontal parallax, as shown in Figure 1a. Actually, when two eyes stare at an object, they converge, so that the object is imaged at the symmetrical point of both eyes’ retinas, producing a fused vision. The associated camera model is depicted in Figure 1b. Between the two cameras, there is rotational variation in addition to translational variation, thus, the pose relationship is extremely difficult to establish, due to the intricate camera motion. The resulting viewpoint and view direction differences seriously limit the matching performance of humanoid-eye binocular images. Recent years have witnessed great progress in image matching algorithms, and these techniques can be broadly classified into two groups: area-based methods and feature-based methods. The area-based methods often depend on appropriate similarity judgment criteria, for creating pixel-level matches between images, including mutual information [4] and normalized cross-correlation coefficient [5]. These approaches are widely used in medical image processing, and achieve favorable performance. However, they are only suitable to image pairs with translation variation, small rotation, and local deformation. Meanwhile, they are also susceptible to appearance changes by noise, varying illumination, and geometric distortions.

Compared with the area-based methods, feature-based ones are often more efficient, and can better handle geometrical deformation. Most feature matching algorithms follow three steps: feature extraction, feature description, and feature matching. Human eyes are more sensitive to edge information, since it is the key to the shape, size, position, and pose of an object. Accordingly, choosing an appropriate corner detector, which can produce adequate edge feature points, is necessary. Among several famous corner detectors [6,7,8,9], the SUSAN detector has better robustness to noise and reduces the location error of edge feature points [10]. Most importantly, the SUSAN operator can extract corner points and edge points at the same time, which retains the contour information of images, to meet the visual characteristic of human eyes focusing on edge points.

A series of methods can be utilized to describe the neighborhood information for SUSAN feature points, the most representative ones are scale invariant feature transform (SIFT) [11] and speeded up robust feature (SURF) [12]. These two algorithms rely on a histogram of oriented gradients for forming float type descriptors, significantly improving the invariance against radiometric transformations. Another form of descriptors is based on the comparison of local intensities, such as BRISK [13] and BEBLID [14], which can speed up the computations and achieve high matching accuracy. Unfortunately, the performance of the aforementioned descriptors in matching SUSAN feature points is not ideal, because of the density of SUSAN features with similar pixel information, and humanoid-eye binocular images with significant viewpoint and view direction differences.

To handle the above-mentioned challenges, we hope to enhance the similarity between the corresponding descriptors, in order to decrease the amount of possible mismatches. Specifically, we try to retain essential information as much as possible when calculating the neighborhood information of feature points. Notably, we find that reducing the image resolution can increase the number of correct matches in humanoid-eye binocular images, thus we pay more attention to establishing multi-scale structures of images. Meanwhile, we argue that computing the neighborhood pixel information of the same feature point at different scales, should enhance the robustness of the matching. Based on the above observation and assumption, a multi-scale descriptor constructing strategy is developed, which computes the main pixel information of each feature point at different scales according to the theory of the SIFT method, by introducing the image pyramid model. Then, these description vectors are combined into a descriptor containing multi-scale information. A series of experiments, with qualitative and quantitative result analyses, have revealed the superiority of our approach over the state-of-the-art.

In summary, our main contributions in this paper include the following three aspects. First, the visual characteristic of human eyes focusing on the edges of objects is introduced into the feature matching process, and the SUSAN operator, which has outstanding edge feature extraction capability, is selected as the feature detector, to address the issue of lacking edge feature points. Second, in response to the problem that mismatches easily exist in humanoid-eye binocular images with significant viewpoint and view direction differences, we propose a novel descriptor, with multi-scale information, for describing SUSAN feature points. Third, a general, yet efficient, refinement strategy is devised, to achieve accurate matching results.

The remainder of this paper is organized as follows. In the next section, we review the work most related to our method. The proposed method is described in Section 3. Later, a series of experiments are carried out in Section 4. Finally, Section 5 presents the conclusions.

## 2. Related Works

Feature matching techniques establish correspondences by comparing characteristics extracted in the local neighborhood of keypoints. In these methods, feature point detectors and descriptors are two important components determining the accuracy. Here, we briefly introduce several feature point detectors and descriptors related to our work.

### 2.1. Feature Point Detectors

Most feature point detectors can be roughly divided into the following two categories in terms of the structure they detect: the blob and the corner [15]. The Laplacian of Gaussian (LoG) detector, was one of the initial blob detectors. Lowe [11] developed a faster detector, to approximate the LoG by Gaussian difference pyramid, which extracts keypoints as the local extrema in the Gaussian scale space. Applying the determinant of the Hessian matrix gives rise to another family of blob detectors, the most well-known of which is the SURF [12] detector. It extracts feature points by the Hessian matrix, together with an integration graph method, thus improving robustness and efficiency. Unlike the circle-like Gaussian response function, Alcantarilla et al. [16] proposed a blob feature detector, the KAZE detector, in which a nonlinear partial differential equation is applied, to perform nonlinear diffusion filtering, thus this detector is edge preserving compared to a Gaussian filter-based detector. However, it suffers from high computation complexity. Another method is WADE [17], which detects feature points by a wave propagation function.

Compared with blob detectors, corner detectors are more suitable to apply to humanoid-eye binocular images, which can meet the needs of edge feature generation with their outstanding edge feature extraction capability.

Corner detectors can be traced back to the work of Moravec [18], he first defined corners as points with low correlation, and calculated the sum-of-squared-differences (SSD) to identify whether the candidate is a corner. Inspired by his work, the famous Harris corner detector [6] was introduced, to address the problems of the Moravec detector, which is not invariant to image rotation and results in high computational complexity. It estimates the autocorrelation by computing first-order derivatives and utilizes the local window to judge whether the gray intensity changes greatly; thus, it has reliable repeatability and distinctiveness. Unfortunately, due to the usage of image derivatives, the majority of the approaches mentioned above are sensitive to noise. To address this challenge, numerous intensity-based methods have emerged. Typically, this category of methods compares the intensity of the surrounding pixels with that of the center pixel to simplify the image gradient computing [19]. Representatively, the SUSAN detector [8] compares the brightness similarity between the local radius region pixels and the nucleus. It improves the robustness to noise, illumination, and rotation variations, due to the use of a circular mask. In recent years, the most popular corner detector has been FAST [9] and its variants [20,21,22]. The FAST family of detectors compare the center pixel to each pixel along a circular ring, and then identify more reliable corner features using machine learning.

### 2.2. Feature Point Descriptors

Once the interest points are detected from raw images, a local patch descriptor is required to be coupled for each feature, in order to establish correspondence across two images. Many feature point descriptors depend on gradient statistics methods for computing description vectors. The SIFT [11] descriptor, highly stable under a variety of image deformations, is the most famous among them. It employs the gradient histogram technique to determine the feature scale and orientation, thus achieving scale and rotation invariance. Tola et al. [23] proposed an effective method, named Daisy, to further enhance the discriminability and reduce computational complexity. It improves the partition strategy by using Gaussian convolution to segment the histogram of gradient direction. Another representative descriptor, SURF [12], significantly accelerates the execution efficiency, by using regional fast summation in the image convolution process. However, the performance of SURF is lower than that of SIFT, under view variation [24].

As already stated, viewpoint and view direction differences are significant in humanoid-eye binocular images. Accordingly, the SIFT descriptor, with robustness to view variation, is an ideal choice to be utilized in feature description. During the last decade, researchers have been racing to find better SIFT-based approaches, to improve matching performance. Arandjelović et al. [25] measured the similarity between SIFT descriptors by using a square root (Hellinger) kernel, instead of the Euclidean distance. It yields superior performance without increasing storage requirements, as the transformation can be done online. Dong and Soatto [26] introduced a simple modification of SIFT, named DSP-SIFT, based on pooling gradient orientations across different domain sizes. To solve the problem of local geometric distortions between image pairs, adaptive binning SIFT (AB-SIFT) [27] adopts an adaptive histogram quantization strategy, to form a description vector. More recently, Cao et al. [28] utilized an increasing homocentric square window, based on the SIFT method, to describe feature points. The resulting descriptor reduces the computational cost, but sacrifices some of the discriminability.

## 3. The Proposed Matching Strategy

In this section, a method to establish correspondence between a pair of humanoid-eye binocular images is explained. The proposed method mainly consists of three steps: feature detection, feature description, and feature correspondence. The details of each step will be described in the following sections.

### 3.1. Feature Point Detection

**Figure 2:**
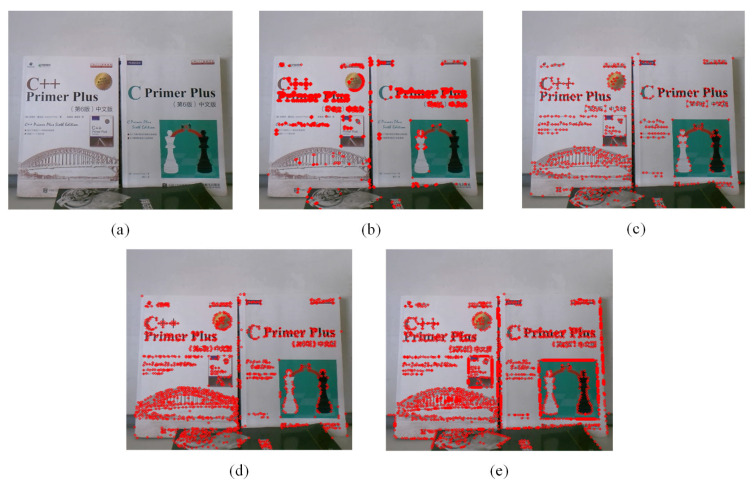
Comparison of four feature point detection methods. (**a**) Input image. (**b**) Feature points detected by Harris. (**c**) Feature points detected by Shi-Tomasi. (**d**) Feature points detected by FAST. (**e**) Feature points detected by SUSAN.

Obtaining enough edge features is the key to imitating the visual characteristic of human eyes being more sensitive to the edge. We compare the performance of four well-known corner detectors: Harris, Shi-Tomasi, FAST, and SUSAN, as shown in Figure 2. The Harris operator produces redundant corners in some local areas of the image, as described in Figure 2b. The Shi-Tomasi and FAST approaches can extract a reasonable amount of corners, but the number of features extracted at some edges is still limited, as depicted in Figure 2c,d, respectively. The SUSAN detector can not only detect enough corners, but also extract plentiful edge features, thus better describing the contour information of the image, as shown in Figure 2e. Due to its good performance, the SUSAN detector is selected to extract feature points in our method.

A circular mask is adopted, to successively process the pixels of the input image in the SUSAN algorithm. The pixel under examination is called the nucleus, and the nucleus is regarded as the center of the mask. In the mask, by calculating the intensity value of the pixels in a neighborhood of the nucleus, the pixels with approximately the same intensity as the nucleus are grouped. These pixels form an area, which is referred to USAN.

**Figure 3:**
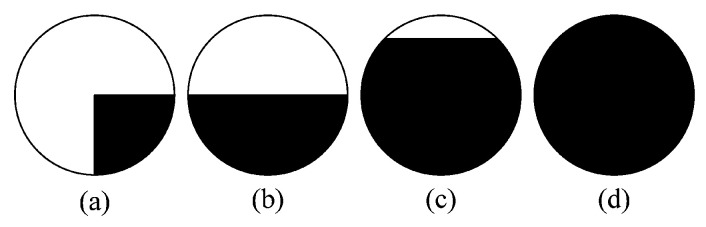
Representative of USAN. (**a**) USAN area reaches the minimum. (**b**) USAN area expands. (**c**) USAN area expands even further. (**d**) USAN area reaches the maximum.

Figure 3 shows a principle diagram of the SUSAN algorithm. The dark area represents the image surface. As depicted in Figure 3a, the USAN area reaches the minimum when the nucleus is located at a corner, and expands even further as the nucleus approaches the surface, as described in Figure 3b,c, respectively. Figure 3d shows that the USAN area reaches the maximum when the nucleus completely lies in the flat region.

Let $c(r,r_{0})$ denote the output of the comparison, where $I(r_{0})$ is the intensity value of the nucleus and I(r) is the intensity value of any other pixel within the mask. A simple equation for determining whether the intensity of a point is the same as that of the nucleus is as follows:

(1) $$c(r,r_{0})=\left\{\begin{array}{cc} 1 & if|I(r)-I(r_{0})|\leq t\\ 0 & if|I(r)-I(r_{0})|>t \end{array}\right\},$$

where *t* is the threshold to decide the similarity. The value of *t* is adaptive, and the optimal value should be provided in terms of different conditions. After comparing the similarity between each pixel and the nucleus within the mask, the USAN area can be specified as:

(2) $$n(r_{0})=\sum_{r\neq r_{0}}^{}c(r,r_{0}).$$

Then, the USAN value is compared with a threshold *g*, which stands for the geometric limit. A point will be regarded as an edge point if its USAN value is less than g. The feature response can be expressed as:

(3) $$R(r_{0})=\{\begin{array}{cc} g-n(r_{0}) & n(r_{0})<g\\ 0 & otherwise \end{array}.$$

Different values of the geometric threshold *g,* result from detecting different features, such as corner and edge. In this paper, the value of *g* is set as three-quarters of the maximum USAN value, which can better detect corners and edge features at the same time.

### 3.2. Feature Point Description

#### 3.2.1. Multi-Scale Structure Establishment

Once the feature points are extracted, designing appropriate descriptors is the second step. To generate multi-scale information, the image pyramid model is introduced into the building descriptors. An image pyramid is a form of multi-scale expression of an image, which arranges the images at different resolutions according to the shape of the pyramid. The bottom image of the image pyramid is an input image to be processed, and the top image is an approximate image with low resolution. It is assumed that the size of the input image is *M* × *N*, the *k*-th layer image of the image pyramid is generated from the *k* − 1-th layer image by Gaussian convolution and down sampling, the *k*-th layer image is then given by:

(4) $$G_{k}(x,y)=\sum_{m=-2}^{2}\sum_{n=-2}^{2}w(m,n)G_{k-1}(2x+m,2y+n),$$

where $G_{k}(x,y)$ stands for the *k*-th layer image. w(m,n) is a 2D function with low-pass characteristics, which is defined as follows:

(5) $$w(m,n)=\frac{1}{256}\left[\begin{array}{ccccc} 1 & 4 & 6 & 4 & 1\\ 4 & 16 & 24 & 16 & 4\\ 6 & 24 & 36 & 24 & 6\\ 4 & 16 & 24 & 16 & 4\\ 1 & 4 & 6 & 4 & 1 \end{array}\right].$$

**Figure 4:**
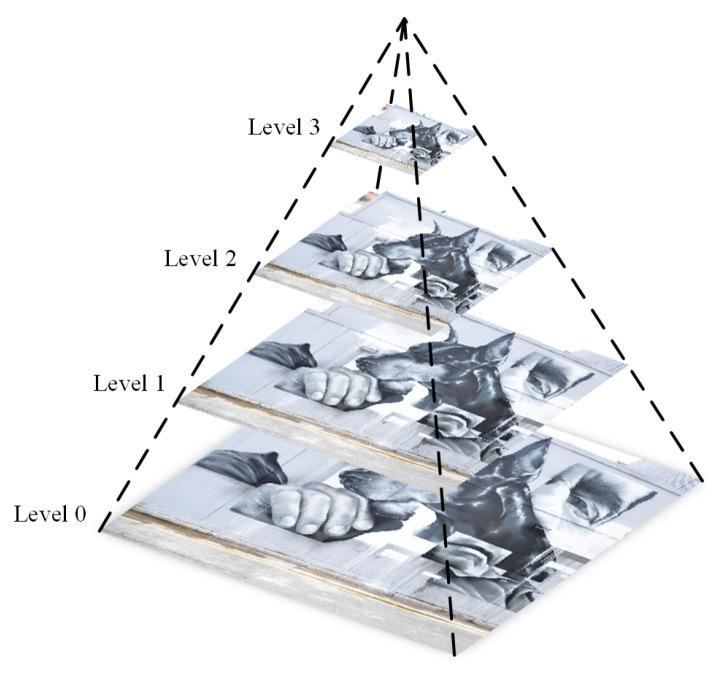
Four-layer image pyramid for feature point description.

A series of images can be generated by iteration, to form a complete image pyramid. In this paper, a four-layer image pyramid is built, as represented in Figure 4.

#### 3.2.2. Main Orientation Assignment Based on Local Gradient Information

The main orientation assignment is an indispensable step for point-based matching methods, to achieve rotation invariance [29]. Therefore, the main orientation for each feature point is computed based on local gradient information of contour points.

Assume L(x,y) is an input image. m(x,y) and θ(x,y) represent the gradient magnitude and orientation, respectively, which are precomputed using pixel differences by the following equations:

(6) $$m(x,y)=\sqrt{{\left(L(x+1,y)-L(x-1,y)\right)}^{2}+{\left(L(x,y+1)-L(x,y-1)\right)}^{2}}$$

(7) $$\theta (x,y)=\tan^{-1}((L(x,y+1)-L(x,y-1))/(L(x+1,y)-L(x-1,y)))$$

The obtained gradient orientations of sample points within a region around the feature point, form an orientation histogram, which has 36 bins, covering the 360 degree range of orientations. In addition, in order to improve the affine invariance of the feature points, a circular window, similar to the SIFT method, is adopted, to weigh the gradient magnitude of each point. The Gaussian-weighted circular window can be represented as follows:

(8) $$w_{i,j}=e^{\frac{-(i^{2}+j^{2})}{2\times 1.5^{2}}}$$

where $w_{i,j}$ denotes the Gaussian-weighted function. (*i*, *j*) represents the relative position between the sample point and the keypoint.

#### 3.2.3. Multi-Scale Descriptor Based on Histogram of Gradient Orientation

**Figure 5:**
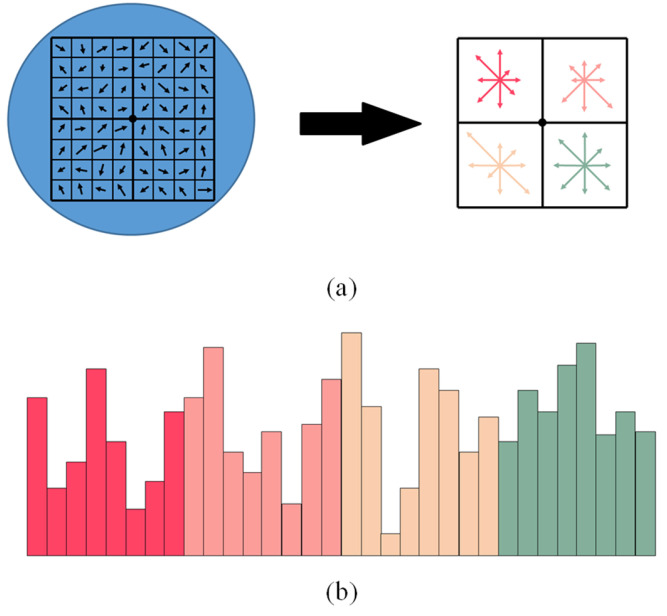
The 32-dimensional descriptor calculated on each layer of the four-layer image pyramid. (**a**) The structure of the descriptor. For the relevant parameters, we follow Lowe’s paper. (**b**) The region around each keypoint is divided into four subregions, each of which contains gradient intensity information in eight directions, forming a 32-dimensional feature vector. Different colors represent the order of feature vectors.

The previous operations have assigned an image location and orientation to each keypoint. The next step is to design a local invariant feature description for feature correspondence. In order to compute the main gradient information of keypoints at different scales, these keypoints are mapped to their corresponding positions of each layer in the four-layer image pyramid. Then, an 8 × 8 region is divided around each keypoint on every layer of image pyramid, and the orientation histograms are determined for feature description, as shown in Figure 5. We can obtain 32-dimensional SIFT descriptors for feature points on each layer image when the other structure parameters are same as SIFT in our experiments.

Compared with the traditional 128-dimensional SIFT descriptor, the resulting 32-dimensional descriptor only retains the gradient information of four subregions around each feature point. This is because viewpoint and view direction differences exist in humanoid-eye binocular image pairs, which may result in significant pixel variation in the region around two corresponding feature points. Therefore, the matching results will be negatively influenced by pixels that are further from the keypoint.

Once four 32-dimensional descriptors for each feature point are obtained, the multi-scale descriptor is constructed. To be specific, these four descriptors are stitched and ordered by decreasing image scales, to form a novel 128-dimensional descriptor with multi-scale information.

### 3.3. Feature Correspondence

The last step of the feature matching algorithm comprises several continuous processes. It starts with matching descriptors, removing non-matches first and further identifying mismatches. The brute-force algorithm is one of the simplest pattern matching algorithms. Because of the large number of comparisons, it sometimes takes more time but is highly precise. The brute-force method compares the descriptor of one feature in the first image to the descriptors of all features in the second image, by using Euclidean distance, their similarity is computed as:

(9) $$d=\sqrt{\sum_{i=1}^{n}{\left(x_{i}-y_{i}\right)}^{2}}$$

where $x_{i}$ and $y_{i}$ denote the descriptors of two feature points. The best candidate match is regarded as two feature points with minimum Euclidean distance for their description vectors.

However, due to the feature points detected by the SUSAN algorithm being numerous and dense, only utilizing the brute-force matching approach results in a large number of mismatches. Therefore, the bidirectional matching strategy is adopted in this paper. The steps of this strategy are briefly introduced as follows:

Step 1: For each feature point $c_{i}$, in the first image, compare its multi-scale descriptor with all the descriptors of feature points in the second image, by computing Euclidean distance. Then, the candidate match is selected with minimum Euclidean distance.

Step 2: Suppose $c_{j}$ is the candidate point for $c_{i}$. Measure the similarity between $c_{j}$ and all the feature points in the first image. If $c_{i}$ is also a candidate point of $c_{j}$, these two points are regarded as a preliminary match.

Notably, many features will not have any correct match, because they are not in the overlapping area of humanoid-eye binocular image pairs with large viewpoint and view direction differences. Therefore, the distance ratio method is employed, to measure the similarity between the obtained preliminary matching pairs in the coarse-matching stage.

In the comparative process, one correct match is defined as:

(10) $$L\leq k\times L_{\max}$$

where k denotes a distance limit, *L* is the Euclidean distance between two descriptors, and $L_{\max}$ is the largest Euclidean distance of all matching pairs. Different values of *k* result in different numbers of matching pairs. As *k* begins to increase, the number of matching pairs increases, but the accuracy of the results decreases. In our method, *k* is 0.6. The number 0.6 is an empirical value, which can effectively identify correct matches.

The accuracy of feature pairs can be greatly improved after the coarse-matching stage, but there are still a few mismatches. Hence, in the refined-matching stage, the RANSAC algorithm [30] is adopted, to further remove the mismatches. Its basic idea is that all matching points are formed into a dataset, and four samples are randomly selected to compute a transformation matrix. Then, the projection error between all the points and transformation matrix is calculated. If the error is less than a threshold *g*, the point is considered to be an interior point. The above steps are repeated and a model is selected with the largest number of interior points as the final transformation matrix, to obtain accurate corresponding pairs.

## 4. Experiments and Discussion

In this section, a series of experiments are conducted to evaluate the proposed method. First of all, the datasets and experimental details used to evaluate this work are presented. Then, we compare the multi-scale descriptor to several popular descriptors and compare their matching results. Finally, the overall performance of the proposed algorithm is evaluated with some of the latest methods.

### 4.1. Datasets and Experiment Details

We evaluate the performance of our multi-scale descriptor against several famous descriptors, on humanoid-eye binocular images and the HPatches [31] dataset. In addition, the overall performance of the proposed work is compared with state-of-the-art algorithms. All the experiments and analyses are implemented on a computer system with Windows 10, a 2.3 GHz processor, and 16 GB memory.

**Figure 6:**
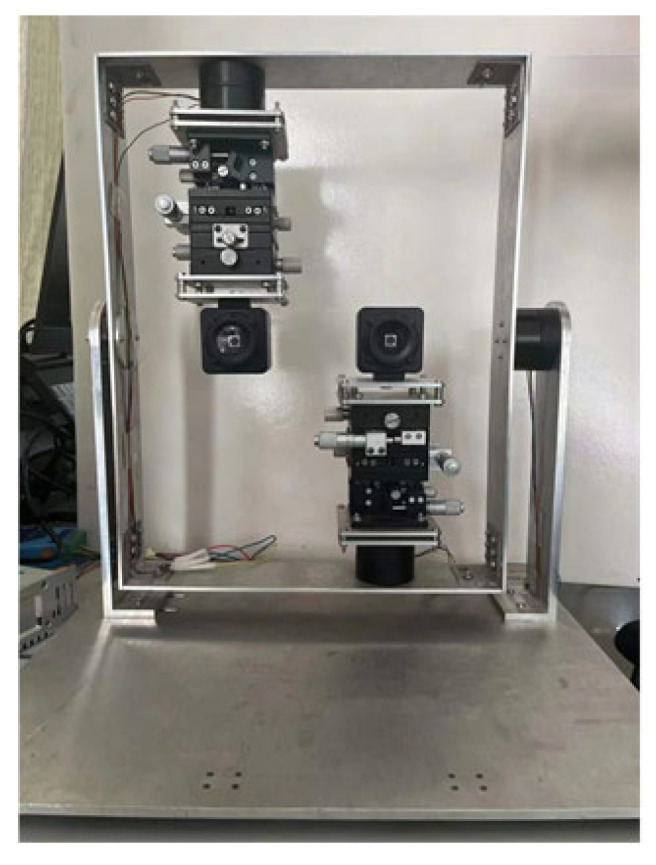
Synergistic perception device imitating human eyes.

**Humanoid-eye binocular images.** To capture humanoid-eye binocular images, we designed synergistic perception equipment, imitating human eyes, as shown in Figure 6. The two cameras are controlled by motors at the bottom, and their centers are constantly on the same horizontal axis. Therefore, these two cameras can rotate left or right at any angle, to simulate human eye imaging.

**HPatches dataset.** The HPatches dataset, is a comprehensive dataset that includes different viewpoints and different illumination scenes, which consists of 116 sequences in total. The HPatches dataset is divided into two subsets; 57 sequences have significant illumination changes with almost the same viewpoint, while 59 sequences have significant viewpoint changes under similar illumination. Each sequence has six images of the same scene. It also provides ground truth homographies between the first image and the remaining five images.

### 4.2. Descriptor Benchmark

**Figure 7:**
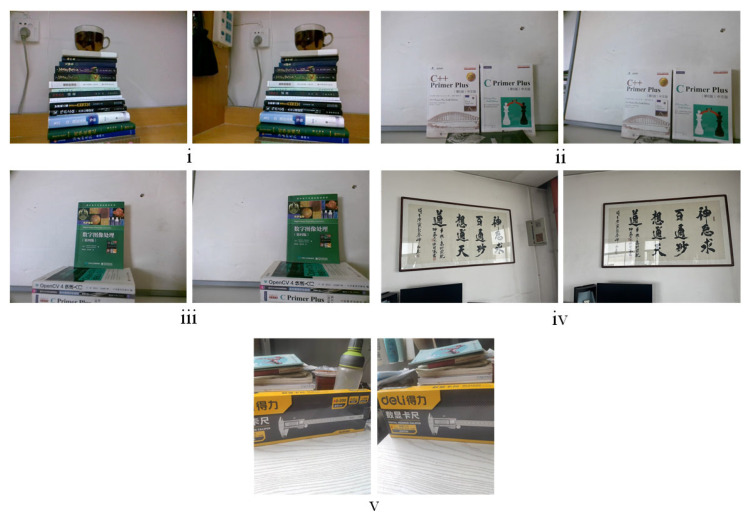
The five pairs of humanoid-eye binocular images used for the descriptor evaluation. (**i**–**v**) are the serial numbers of test image pairs.

**Figure 8:**
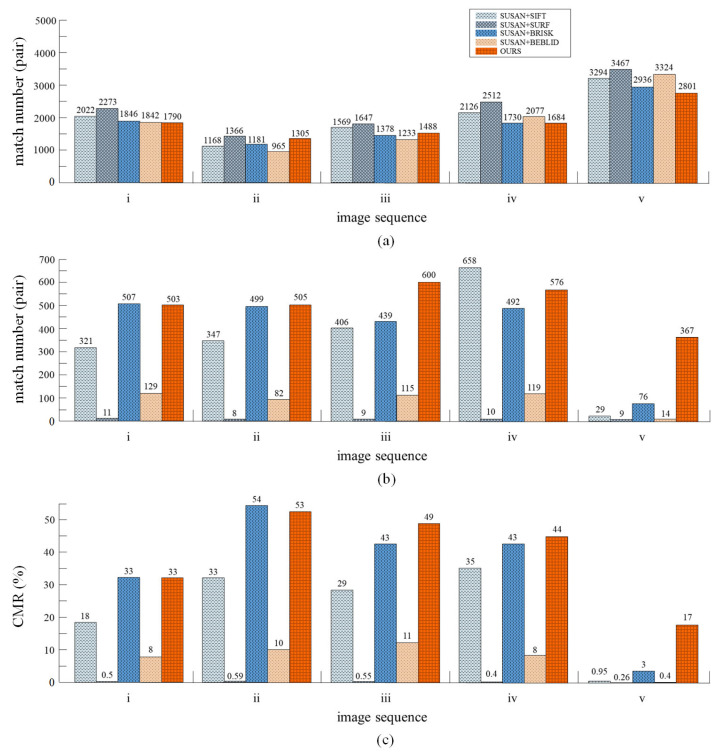
Comparison of the performances of different descriptors on humanoid-eye binocular images. (**a**) Total number of matching pairs. (**b**) Number of matching pairs after the refined-matching stage. (**c**) Correct matching rate.

The performance of the proposed multi-scale descriptor is evaluated against some of the most relevant methods such as SIFT, SURF, BRISK, and BEBLID. As already mentioned, matching experiments of descriptors, on the humanoid-eye binocular images captured by our equipment, were carried out, some of the test images are shown in Figure 7. For fairness, the SUSAN detector is used in the feature point detection, and the subsequent removal procedures are consistent with our method. The evaluation index *CMR* (correct matching rate), is utilized to evaluate the proposed descriptor. The definition of *CMR* is as follows:

(11) $$CMR=\frac{N_{r}}{N_{c}}$$

where $N_{r}$ is the number of matches in the refined-matching stage and $N_{c}$ is the number of matches in the coarse-matching stage. With this metric, a higher *CMR* value corresponds to a better matching quality. The comparison of the results is displayed in Figure 8.

Based on the results, it can be seen that, although the total number of feature pairs generated by the multi-scale descriptor (proposed work) is somewhat less than with other descriptors, more correct matching pairs can be produced in the final matching results. The multi-scale descriptor can generate an average of 510 accurate corresponding pairs, among five pairs of test images. The second highest average is BRISK and the third highest average is SIFT, which generate an average of 403 feature pairs and 352 feature pairs, respectively. The results in Figure 8c, show that the multi-scale descriptor produces the highest average CMR, with 39.2% in all image sequences. The second highest average is BRISK, followed by SIFT, BEBLID, and SURF. Specifically, the multi-scale descriptor obtains a higher average CMR, than the BRISK and SIFT descriptors, with 4% and 16.01%, respectively.

As mentioned above, the proposed method reduces the total number of matches, as well as the number of incorrect matches. This is primarily due to the introduction of the image pyramid model when constructing descriptors, which generates the multi-scale information for descriptors, to significantly enhance the anti-interference ability of matching and restrict the matching between unreliable feature points. The results show that, our method is capable of increasing the number of accurate corresponding pairs in humanoid-eye binocular images.

Besides comparing the *CMR* values of the above five methods, the second evaluation of the proposed descriptor is to use the metric known as recognition rate [15], on the HPatches dataset. It can be computed following these steps:

(1) The SUSAN detector is used to extract *N* points in the first image of an image sequence in all tests. Then, these *N* points are projected to the remaining five images.
(2) Matching the first image with the remaining five images in each image sequence. To adjust the number of matches, the precision, $p=\frac{TP}{MP}$, is considered, where *TP* is the number of correct matches and *MP* is the number of constructed matches.
(3) The recognition rate is defined as: $r=\frac{TP}{N}$ [15]. It indicates the ratio of the number of correct matches to the number of feature points.

**Figure 9:**
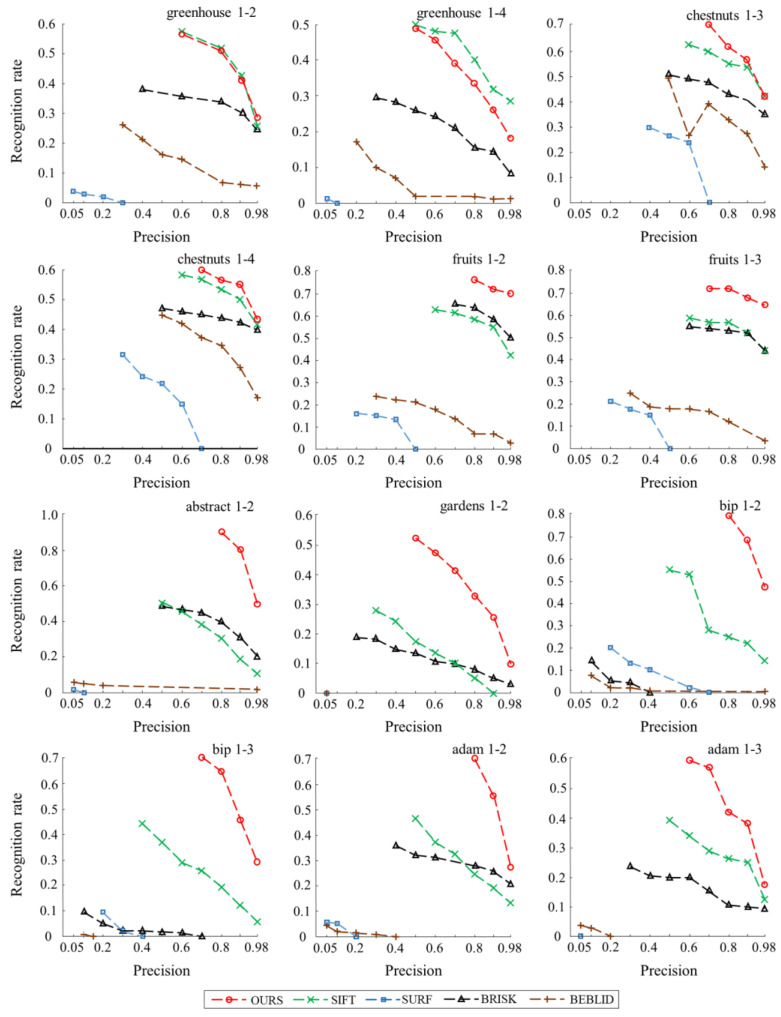
Descriptor recognition rates of several methods.

The plots in Figure 9 show the recognition rate curves for each descriptor on seven image sequences chosen from the HPatches dataset. Based on these comparison results, in three image sequences (greenhouse, chestnuts, fruits), where illumination change exists, the recognition rate of the multi-scale descriptor (proposed work) is better than other descriptors, except in the greenhouse sequence. In four image sequences (abstract, gardens, bip, adam), where significant viewpoint change exists, our descriptor outperforms all others under different precision.

It is worth noting, that having a high recognition rate is not always beneficial. A higher recognition rate leads to lower precision, which produces an excessive number of mismatches. In this case, it can be observed that the lowest precision produced by the multi-scale descriptor is higher than that of the other descriptors in all image sequences. The average lowest precision is increased by 0.16 and 0.3, compared to the second best (SIFT) and third best (BRISK) descriptors. In other words, our approach can maintain high accuracy even when the recognition rate is high.

The other descriptors perform less well, especially SURF and BEBLID, possibly because (1) the performances of the descriptors are influenced by the SUSAN detector, and (2) we do not perform any data training for these approaches, and merely use the functions in the OpenCV library when testing descriptors.

Finally, the run time of the proposed multi-scale descriptor and other descriptors mentioned above are measured on the humanoid-eye binocular images, as shown in Table 1. These values have been averaged for five pairs of humanoid-eye binocular images. Compared with other famous descriptors, the multi-scale descriptor requires more calculation time than others, since it is computed on four different scales, while other descriptors are constructed on a single scale. In future research, we will investigate how to reduce the computational cost.

### 4.3. Evaluation of the Overall Algorithm

For evaluating the overall performance of the proposed algorithm, three state-of-the-art methods: GMS [32], SuperGlue [33], and DFM [34], are chosen for comparison. GMS is a method that converts motion smoothness into statistical problems, to identify false matches, significantly improving efficiency and robustness. SuperGlue, a deep learning technique, is able to reason about the underlying 3D scene and feature assignments jointly, by using a graph neural network. DFM aligns images with coarse geometric transformation estimates, and then applies a coarse-to-fine strategy, to achieve better localization and matching performance.

The overall performance of the proposed method is measured by the pixel-wise distances between the matched features and their ground truth projections on the pair images. Homography matrix estimation tasks are conducted on humanoid-eye binocular images. For these tasks, following Xia’s work [35], homography error is considered, which adopts RANSAC as the estimator to derive the geometric model. In this case, a corresponding estimated homography is identified as accurate when its homography error is less than three pixels, and the accuracy (Acc.) is utilized as the evaluation metric for geometric estimation, which represents the ratio of accurate estimates to all estimates. In addition, the specific projection errors (Proj.) are also reported, for intuitive analysis. These values have been averaged for all test images. The quantitative results regarding the four compared methods are presented in Table 2.

Based on these results, our method has the best performance on two metrics (Acc. and Proj.), producing the highest accuracy and the lowest projection error, 96.13 and 1.63, respectively. These results demonstrate the superiority of our approach relative to other state-of-the-art feature matching algorithms.

To further exploit the practical value of our method, the HPatches dataset is used for testing. The pixel distance errors between estimated and ground truth projections of the points are calculated as three evaluation criteria: maximum, mean, and variance. These values have been averaged for the HPatches dataset. We report the results of these three metrics on image sequences with illumination changes and image sequences with viewpoint changes, as shown in Table 3.

As the comparison results in Table 3 show, compared with other algorithms, the proposed method achieves the best matching accuracy in both image sequences (illumination changes and viewpoint changes). From the fourth and seventh columns of Table 3, it can be seen that our method produces the lowest variance of distance errors, which means that it can effectively remove the majority of incorrect matching pairs and significantly improve the matching stability.

**Figure 10:**
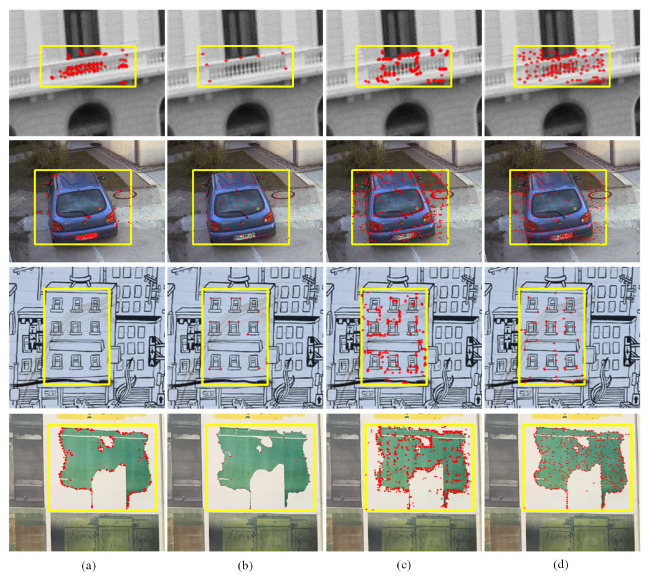
Visual results of several methods. (**a**) GMS. (**b**) SuperGlue. (**c**) DFM. (**d**) Ours.

In addition to quantitative analysis, we also performed visual tests on the HPatches dataset. Figure 10 shows the positions of feature points from matching pairs on reference images. From the yellow boxes marked in Figure 10, the distribution of feature pairs can be analyzed more intuitively. Compared to GMS and SuperGlue, the proposed method produces more corresponding pairs, especially edge pairs, significantly enhancing the ability to describe image outline information. Note that, although DFM generates the most matching pairs, the majority of these are clustered together, and so have little impact on the feature matching. This is due to the fact that, for some textures of an image, only a few features are enough to describe the details, and redundant features inevitably increase the computational complexity. On the contrary, the matching pairs produced by our method are evenly distributed. It not only obtains sufficient edge features but also extracts feature points, to describe details in the flat area, which can better satisfy the characteristics of human vision. Based on these results, it is shown that the proposed method is competitive with, and better than some of, the state-of-the-art algorithms.

## 5. Conclusions

Aiming at the visual characteristic that human eyes are more sensitive to edge information, we propose a matching method, based on the SUSAN–SIFT algorithm, for humanoid-eye binocular images. To detect corner points and edge points at the same time, we conduct comparisons among several popular detectors and select the SUSAN operator for feature detection. On this basis, the novel descriptor, with multi-scale information, is proposed for feature description. To improve the matching accuracy, refinement procedures are designed to remove mismatches. The results, based on quantitative and qualitative analyses, show that the proposed approach is capable of retaining edge information and improving the accuracy of corresponding pairs, compared with several state-of-the-art methods.

Even though our method has shown significant advantages over several state-of-the-art methods, in terms of the effectiveness of producing edge corresponding pairs, accuracy of feature matching, and robustness to illumination variations, it has a worse computational efficiency compared to other methods. This is because the proposed multi-scale descriptor is computed on four different scales, while other descriptors are constructed on a single scale. Consequently, how to construct descriptors on a single scale with main gradient information will be investigated in further research, which can reduce calculation time.

## Figures and Tables

**Table 1:** The computational time of comparative experiments.

Method	Environment	Run Time (s)
SUSAN + SIFT	i5-6300HQ CPU @2.30 GHz (Visual Studio 2019)	153
SUSAN + SURF	i5-6300HQ CPU @2.30 GHz (Visual Studio 2019)	86
SUSAN + BRISK	i5-6300HQ CPU @2.30 GHz (Visual Studio 2019)	27
SUSAN + BEBLID	i5-6300HQ CPU @2.30 GHz (Visual Studio 2019)	30
Ours	i5-6300HQ CPU @2.30 GHz (Visual Studio 2019)	154

**Table 2:** The comparison of homography estimation on humanoid-eye binocular images.

Method	GMS	Super Glue	DFM	Ours
Acc.	78.78	59.97	82.39	96.13
Proj.	11.24	6.21	3.65	1.63

**Table 3:** Results of the pixel distance errors on the HPatches dataset.

Methods	Illumination Changes			Viewpoint Changes		
	Maximum	Mean	Variance	Maximum	Mean	Variance
GMS	61.98	2.02	40.24	200.20	5.5	2754.04
SuperGlue	88.19	2.25	111.27	37.21	3.39	27.68
DFM	25.20	0.65	1.16	114.99	1.96	297.68
Ours	3.90	0.65	0.61	7.29	1.67	1.20

## Data Availability

Data will be made available on request.
